# Polish Validation of the SarQoL^®^, a Quality of Life Questionnaire Specific to Sarcopenia

**DOI:** 10.3390/jcm7100323

**Published:** 2018-10-04

**Authors:** Jerzy Konstantynowicz, Pawel Abramowicz, Wojciech Glinkowski, Ewa Taranta, Ludmila Marcinowicz, Malgorzata Dymitrowicz, Jean-Yves Reginster, Olivier Bruyere, Charlotte Beaudart

**Affiliations:** 1 Department of Pediatrics, Rheumatology, Immunology, and Metabolic Bone Diseases, Medical University of Bialystok, 15-274 Bialystok, Poland; pabram@o2.pl; 2 Polish Telemedicine Society, Center of Excellence “TeleOrto” for Telediagnostics and Treatment of Injuries and Disorders of the Locomotor System, 02-091 Medical University of Warsaw, Warsaw, Poland; w.glinkowski@gmail.com; 3 Department of Primary Health Care, Medical University of Bialystok, 15-054 Bialystok, Poland; taranta59@wp.pl (E.T.); ludmila.marcinowicz@umb.edu.pl (L.M.); 4 Rehabilitation Department, Central Military Medical Clinic, 00-911 Warsaw, Poland; malgorzata.reh.lecz@interia.pl; 5 Department of Public Health, Epidemiology and Health Economics, University of Liège, 4000 Liège, Belgium; jyr.ch@bluewin.ch (J.-Y.R.); olivier.bruyere@ulg.ac.be (O.B.); c.beaudart@uliege.be (C.B.); 6 World Health Organization Collaborating Center for Public Health Aspects of Musculoskeletal Health and Aging, University of Liège, 4000 Liège, Belgium

**Keywords:** sarcopenia, quality of life, translation, questionnaire-based research

## Abstract

Recently, SarQoL^®^ (Sarcopenia and Quality of Life), a quality of life (QoL) questionnaire specific to sarcopenia, was successfully developed. For practical reasons, there is a great interest in validating this questionnaire in other populations. The aim of this cross-sectional study was to translate and adjust the SarQoL^®^ into Polish and to standardize the validity of this method for the assessment of sarcopenic individuals in Poland with regard to psychometric properties. The English version was used for the translation process. A total of 106 community-dwelling Caucasian subjects aged 73.3 ± 5.94 years (65.1% females) were studied, with 60 participants being diagnosed sarcopenic. The translation and cross-cultural adaptation was carried out in five phases according to specific standard guidelines. There were no major linguistic issues in the translation process. The data confirmed a good discriminant validity, i.e., significantly lower scores for all domains (reduced global QoL in sarcopenic subjects compared to non-sarcopenic ones; 54.9 ± 16.5 vs. 63.3 ± 17.1, *p* = 0.013), and high internal consistency (Cronbach’s alpha coefficient was 0.92). The significant correlation of the SarQoL^®^ scores with those of other questionnaires (SF-36v2^®^ Health Survey and EuroQoL-5-Dimension) that are supposed to have similar dimensions indicated the consistent construct validity of the SarQoL^®^-PL questionnaire. No floor/ceiling effects were found. An excellent agreement was found between the test and the re-test (intraclass coefficient correlation (ICC): 0.99). The first Polish version of the SarQoL^®^ questionnaire is valid and consistent and therefore may be used with reliability for clinical and research purposes regarding QoL assessment of sarcopenic individuals. However, further research, in particular prospective studies, is needed to determine potential limitations and the suitability of the new tool for the Polish scenario and specificity.

## 1. Introduction

Sarcopenia is regarded as a progressive decrease of skeletal muscle mass and function with ageing. The first definition of sarcopenia was developed by Rosenberg, and initially only muscle mass was incorporated [1]. Subsequent definitions included decreased muscle function, since in some epidemiological studies a higher decline in muscle strength than in mass has been observed [2]. At present, different approaches to the definition of sarcopenia with a variety of cut-off values and characteristics are proposed; however, there is a consensus that loss of both muscle mass and muscular function (strength or physical performance) is required to define this condition [3,4,5,6]. Sarcopenia is apparently associated with various health consequences and outcomes, such as physical impairment, increased risk of falls, fractures, hospitalization rate, depression, and mortality and is considered an important public health issue for aging societies in the context of demographic decline [7,8,9,10,11]. The majority of these age-related conditions resulting from sarcopenia may potentially impact quality of life and self-esteem. The associations between sarcopenia and quality of life in older populations are being increasingly studied. Recently, SarQoL^®^ (Sarcopenia and Quality of Life), a quality of life (QoL) questionnaire specific to sarcopenia, was developed and validated [12]. This novel, self-administrated, multidimensional questionnaire designed for community-dwelling elderly subjects aged 65 years and older is composed of 55 items, which are translated into 22 questions rated on a four-point Likert scale, and includes seven major domains of dysfunction concerning the condition of sarcopenia: physical and mental health, locomotion, body composition, functionality, activities of daily living, leisure activities, and fears. The questionnaire is scored out of 100 points using a special scoring algorithm, with higher scores reflecting a better quality of life (QoL). SarQoL^®^ was initially developed and validated in French, but the translated English and Romanian versions of the questionnaire have been recently validated and have been proven to be understandable, valid and consistent versions of the questionnaire [13,14]. These analyses of SarQoL^®^ have also demonstrated its ability to discriminate sarcopenic subjects from non-sarcopenic ones based on their health-related QoL. 

Transcultural adaptation and compatibility studies of the questionnaire instrument need to be done to ensure its cultural equivalence and usefulness in different populations. In general terms, a mere technical translation from an original version into other languages does not seem to be sufficient to ascertain the validity of the instrument for clinical purposes. Translated versions of the questionnaire should be validated and standardized. As there was a gap in the methodological approach for this in Poland, we attempted to address this issue by adjusting the new tool to the Polish population. The objectives of this study were to translate the SarQoL^®^ questionnaire into Polish and to evaluate its main psychometric properties, i.e., discriminative power, validity, reliability, and floor/ceiling effects.

## 2. Material and Methods

### 2.1. Participants and Protocol

A total of 106 community-dwelling elderly subjects (aged 65 years and older) were recruited in two outpatient clinics in Poland (Bialystok and Warsaw), both of which had large proportions of older and geriatric patients. All participants were informed about the objectives and procedures, and then written informed consent was obtained. Prior to commencing the recruitment process, older people assigned to the clinics were provided with concise printed educational material addressing major geriatric problems and sarcopenia. The study was approved by the Ethics Committee of the Medical University of Bialystok (Poland), approval No. R-1-002/126/2017. Participants with an amputated limb, or who were immobilized, had active malignancy, or mental illness, and who were unable to cooperate, understand and/or complete the questionnaires were excluded. General variables analyzed included age, gender, and educational and marital status. Anthropometric parameters (body mass, height, and waist, hip and thigh circumferences) and blood pressure were measured using standard methods by trained examiners. Sarcopenia was diagnosed according to the approach of the European Working Group on Sarcopenia in Older People (EWGSOP) [3]. The EWGSOP recommends using the presence of both decreased muscle mass and decreased muscle function (strength). The study was performed in two centers and, due to limited access to dual-energy X-ray absorptiometry (DXA) equipment, skeletal muscle mass (SMM) was estimated using a special formula derived from the Lee equation [15]: (0.244 × body mass) + (7.8 × height) + (6.6 × sex ratio) − (0.098 × age) + (ethnicity ratio − 3.3). Muscle strength was evaluated as handgrip strength using a hydraulic hand dynamometer (Saehan SH5001) with cut-off values of <20 kg for women and <30 kg for men. The equipment was calibrated prior to conducting the study. The measurements were performed twice on the dominant hand, and the mean value was used for further analyses.

### 2.2. Procedures

#### 2.2.1. Polish Translation

The translation and cross-cultural adaptation went through five phases according to specific guidelines [16]: (1) two independent translations from English to Polish by two bilingual translators who were Polish native speakers, one of whom had a medical background, and the other being a novice regarding the topic; (2) synthesis of the initial translations to provide a single translated “version 1”; (3) two independent reverse translations into English, blind to the original version of the SarQoL^®^, by translators having English as their first language and no medical background; (4) an expert committee review to compare the reverse translations with the original questionnaire, resulting in a pre-final “version 2” of the Polish translation of the questionnaire and a report of the discrepancies; (5) test of the pre-final “version 2” on 10 sarcopenic subjects to ensure understanding of the purpose and meaning of each question, leading to the final version of the SarQoL^®^-PL.

#### 2.2.2. Psychometric Validation of the Polish Version of the SarQoL^®^

Validation of the psychometric properties of the SarQoL^®^-PL consisted of assessment of its discriminative power, internal consistency, and potential floor and ceiling effects, followed by determination of the construct validity and test-retest reliability according to recommendations proposed by Terwee et al. [17]. The construct validity and test-retest reliability were carried out on sarcopenic subjects.

- Discriminative power. The studied group was divided into sarcopenic and non-sarcopenic subjects based on the definition described above. We assumed that QoL is worse in sarcopenic subjects compared to subjects without a diagnosis of sarcopenia. An independent sample *t*-test was performed to assess the difference of overall and domain QoL scores between sarcopenic and non-sarcopenic subjects.
- Internal consistency. To measure internal consistency, understood as an estimation of the questionnaire’s homogeneity, Cronbach’s alpha coefficient was applied [18]. A coefficient value greater than 0.70 indicates a high level of internal consistency. By deleting one domain at a time, each domain’s impact on reliability was also considered. The correlation of each domain with the total score of the SarQoL^®^-PL was also assessed using Pearson´s correlations, since scores were normally distributed. A correlation above 0.81 is considered excellent, one between 0.61 and 0.80 very good, and one between 0.41 and 0.60 is regarded as good.
- Floor and ceiling effects were defined as when a high percentage of the population had the lowest or the highest score, respectively. Floor and ceiling effects higher than 15% were considered to be significant.
- Construct validity. The construct validity was investigated by measuring the convergent validity, and this analysis was performed only with sarcopenic participants. Apart from completing the SarQoL^®^-PL questionnaire, subjects were asked to complete two other commonly accepted questionnaires that were thought to have similar dimensions:
  1. The generic Short Form-36 Health Survey questionnaire (SF-36v2®) [19], which contains 36 items gathered into eight health domains: physical functioning, role limitation due to physical problems, pain, vitality, general health, role limitation due to emotional problems, mental health, and social functioning. The total raw score computed for each health domain scale (i.e., from 0, reflecting the worst QoL, to 100—the best QoL) was aggregated with the use of Health Outcomes Scoring Software 5.0 into a physical component summary (SF-36v2^®^ PCS) and mental component summary (SF-36v2^®^ MCS), providing reliable and valid summaries of a respondent’s physical and mental status.
  2. The EuroQoL 5-dimension (EQ-5D) questionnaire [20] includes five domains: mobility, usual activities, self-care, pain/discomfort, and anxiety/depression, as well as the EQ Visual Analogue Scale (EQ-VAS), as a measure of overall self-rated health status. Each of the 5 dimensions comprising the EQ-5D was divided into 5 levels of perceived problems, from level 1—indicating no problem, to level 5—indicating extreme problems. A unique health state was defined by combining the reported level from each of the five dimensions and referring the result to a 5-digit code, which was subsequently converted into a single index value (EQ-5D index value).

Spearman’s correlations were used to measure the correlation between the total score of SarQoL^®^-PL and the scores of the other questionnaires, as the data of the SF-36v2 and the EQ-D5 questionnaires were not normally distributed.

- Test-retest reliability. To analyze the test-retest stability of our Polish version of the SarQoL^®^, the sarcopenic participants were asked to fill in the questionnaire once again after a two-week interval. The reliability was assessed by means of an intraclass coefficient correlation (ICC). An ICC over 0.7 is considered an acceptable reliability. Participants were also questioned about having any change in their general health (physical and mental) during the preceding two weeks. Only the results of the subjects who did not report any significant health change over that two-week period were included in the analysis.

### 2.3. Statistical Analysis

All of the analyses described below were carried out using IMB SPPS Statistics 21.0. Results were considered statistically significant at the 5% critical level (*p* < 0.05). Normality of quantitative variables was tested by the Shapiro–Wilk test. Quantitative variables with a normal distribution were expressed as a mean ± SD, quantitative variables which showed a non-normal distribution were expressed as a median (P25–P75), and qualitative variables were reported as absolute and relative frequencies (%). Differences in characteristics between sarcopenic and non-sarcopenic subjects were tested with the parametric Student’s *t*-test or the non-parametric Mann–Whitney *U* test for quantitative variables, and with a χ^2^ test for qualitative variables. For internal consistency, the Cronbach alpha coefficient was applied. The correlation of each domain with the total score of the SarQoL^®^-PL was determined using Pearson´s correlation coefficient, since data from the SarQoL^®^-PL were normally distributed, whereas the correlation between the SarQoL^®^-PL and the other questionnaires (the data of which were not normally distributed) was evaluated using Spearman’s correlation coefficient.

## 3. Results

### 3.1. Descriptive Analyses

A total of 106 subjects (mean age 73.3 ± 5.94 years) were recruited for this study, of which 65.1% were women. Among them, 60 (56.6%) were diagnosed with sarcopenia (43 women and 17 men). Sarcopenic subjects were older than non-sarcopenic ones (78.4 ± 8.05 vs. 71.3 ± 5.24 years, *p* = 0.003). Analyses revealed a difference between the groups in relation to anthropometric traits, with sarcopenic subjects demonstrating significantly lower weight than non-sarcopenic ones (*p* = 0.03), and lower waist and thigh circumferences (*p* = 0.023 and 0.015, respectively). Sarcopenic subjects also presented evidently reduced muscle strength (19.0 kg (17.0–23.0) vs. 31.0 kg (25.0–41.2); *p* < 0.001), as well as lower systolic blood pressure (136.8 ± 18.1 vs. 145.6 ± 22.9 mmHg; *p* = 0.03). No differences in height, body mass index (BMI), hip circumference, waist-to-hip ratio, or diastolic blood pressure were found between the groups. There were no differences regarding sex, marital status, educational status, number of sustained falls, number of fractures, number of coincident diseases, or number of drugs consumed either. All demographic, anthropometric and social characteristics are presented in Table 1.

### 3.2. Translation Process 

We faced no major difficulties during the translation process. All 22 questions of the original SarQoL^®^ questionnaire were translated flawlessly, without major problems. However, some minor discrepancies were found, reflecting the cultural context or semantic issues specific to the Polish language. The pre-test was initially conducted on 10 subjects. Minor changes were subsequently included in the pre-final version, but these modifications altered neither the meaning of the sentences nor the essence. Certain linguistic amendments were mainly related to phrasing, wording used for the 4-Likert scale choices, and terminology convention.

### 3.3. Psychometric Validation Analyses

• Discriminative power

Sarcopenic subjects reported a reduced global quality of life compared to non-sarcopenic subjects (54.9 ± 16.5 versus 63.3 ± 17.1, *p* = 0.013). The discriminant validity of the questionnaire was thereby confirmed. The domains of physical and mental health (D1), locomotion (D2), body composition (D3), and activities of daily living (D5) were also significantly lower in sarcopenic subjects compared to non-sarcopenic ones. Other domains (D4, D6, and D7) did not differ between groups (Table 2).

• Internal consistency

A Cronbach’s alpha of 0.92 was found, indicating a high internal consistency. Deleting the domains one at a time led to Cronbach’s alpha values varying between 0.894 (when deleting domain D2 locomotion) and 0.944 (when deleting domain D6 leisure activities). Moreover, all domains showed a significant positive correlation with the total score of the SarQoL^®^-PL, ranging from *r* = 0.46, *p* < 0.001 (for domain D6 leisure activities) to 0.94, *p* < 0.001 (for domain D2 locomotion) (Table 3).

• Floor and ceiling effects

No subjects presented with the lowest score in the questionnaire (0 points) or the maximum score (100 points). Therefore, neither floor nor ceiling effects were found for the questionnaire.

• Construct validity

The construct validity was investigated by measuring the convergent validity. Correlation between the SarQoL^®^-PL questionnaire and the SF-36v2^®^ and EQ-5D questionnaires, which were supposed to have similar dimensions, were assessed. As expected, the SarQoL^®^-PL questionnaire showed a strong and significant correlation with the SF-36v2^®^ MCS, SF-36v2^®^ PCS, EQ-5D index value, and EQ-VAS, ranging from *r* = 0.62, *p* < 0.001 for SF-36v2^®^ MCS to *r* = 0.88, *p* < 0.001 for SF-36v2^®^ PCS. Results of the construct validity assessment are presented in Table 3.

• Test-retest reliability

Thirty-eight subjects agreed to complete the SarQoL^®^-PL after a two-week interval. The global score of the SarQoL^®^-PL moved from 65.1 ± 13.4 to 65.3 ± 13.5, which reflected the stability of the questionnaire across time. Excellent agreement was found between the test and the retest (Table 4). For both the total score and for the individual domains of the SarQoL^®^-PL, a very high ICC was found (from 0.96 for D7 to 1.00 for D6—reflecting perfect reliability).

## 4. Discussion

This study was conducted to provide a convenient and reliable instrument for quality of life assessment among elderly Polish individuals, particularly those who are affected by sarcopenia. The original version of SarQoL^®^ was first developed and validated in French, followed by the English and Romanian versions. To date, the questionnaire is available online (www.sarqol.org) in 21 languages, while another 15 translations are currently in progress. Our study provides validation of the SarQoL^®^ in a different population, since the demographic characteristics in Poland are slightly different compared to other countries. Using a complex, rigorous, multistage translation and cross-cultural adaptation of the English version of the questionnaire, we attempted to develop the first Polish translation of the SarQoL^®^. Evidence for equivalence between the Polish and English versions of the questionnaire was provided by SarQoL^®^-PL’s consistency with the original and by its high internal consistency (Cronbach’s alpha of 0.92), which appeared comparable with the original version. Moreover, the SarQoL^®^-PL underwent a validation process in which it demonstrated appropriate psychometric properties for clinical and research applications. 

We collected a relatively large cohort of older individuals with sarcopenia, and this was presumably due to the specific demographic nature and distribution of the studied populations at the two sites. It is also possible that some participants may have been more aware of their risk of sarcopenia, as the target population had already been informed and was knowledgeable regarding the disease at the invitation stage. Nevertheless, even if our large cohort was due to the intentional and purposeful manner of recruitment, this does not detract from the main findings reported in this study. The sarcopenic subjects presented a reduced global QoL compared to the non-sarcopenic subjects. The discriminant validity of the Polish version of the questionnaire was thereby confirmed. Similar results were observed in the other translated versions of the SarQoL^®^ [13,14]. In our study, the domains of physical and mental health (D1), locomotion (D2), body composition (D3), and activities of daily living (D5) were also significantly lower in sarcopenic subjects compared to non-sarcopenic ones. For the domains D4 (functionality) and D7 (fears), the questionnaire’s discriminative power showed differences between sarcopenic and non-sarcopenic groups; however, these were not statistically significant. This can be at least partly explained by the small sample size. There were no differences between the groups within domain D6 (leisure activities), which might be elucidated by the cultural context in part, i.e., older persons in Polish society may be generally less involved in pastimes, live entertainment and also, importantly, outdoor activity. 

The construct validity was investigated by measuring the convergent validity. As expected, the SarQoL^®^-PL questionnaire showed a strong and significant correlation with some domains of the SF-36v2^®^. Good correlations were also found between the total score of the SarQoL^®^-PL questionnaire and the EQ-5D index value and EQ-Visual Analogue Scale. These correlations support the consistent construct validity of the SarQoL^®^-PL. Finally, an excellent agreement was found between the test and the re-test, which was even higher than that observed for the English translation. The SarQoL^®^-PL seems to be stable across time when no health changes occurred. Comparison between versions in three different languages revealed similar correlations of the total score of the SarQoL^®^ questionnaire with its individual domains (Table 5). Noticeably, this consistency of results forms an excellent foundation for discussion and debate on future multi-population studies using this method. Therefore, the implementation of this novel, language-specific tool in the Polish geriatric population may presumably assist general practitioners in early identification of individuals with an unsatisfactory quality of life, lower performance, or relevant expectations regarding lifestyle and health care. The Polish version of the questionnaire may also rationalize future intervention studies on quality of life in the aging population. 

In our study, the sarcopenic subjects had a significantly lower weight than non-sarcopenic ones (*p* = 0.03), lower waist circumference (*p* = 0.023) and lower thigh circumference (*p* = 0.015). Interestingly, some reports suggest a low thigh circumference is associated with an increased risk of developing premature morbidity and mortality. This adverse association may be related to a reduced muscular mass in the anatomical region [21]. Sarcopenic subjects also presented considerably reduced muscle strength (19.0 vs. 31.0 kg; *p* < 0.001), as well as lower systolic blood pressure (136.8 ± 18.1 vs. 145.6 ± 22.9 mmHg; *p* = 0.03). No difference regarding sex, height, BMI, hip circumference, or waist-to-hip ratio were observed, and neither were differences in marital and educational status, number of previous falls and fractures, comorbidities, pharmacological medication, or diastolic blood pressure found between groups.

The major limitation of our study was related to the different method of muscle mass assessment. We were not able to determine appendicular muscle mass with the use of the DXA method; the study was conducted in two different centers with very limited access to the DXA body composition equipment in one of them. In our study group, muscle mass was estimated using the Lee et al. equation (described above). We are aware that anthropometric measurements are prone to errors and may produce pitfalls, and thus they are not recommended for routine use according to the EWGSOP [3,4]. Nevertheless, the equation provided by Lee et al. for estimation of skeletal muscle mass has been previously validated and proved to have a high level of agreement with DXA-predicted measurement (*kappa* 0.743; *p* < 0.001), as well as a high sensitivity (86%) and specificity (89%) [22]. 

## 5. Conclusions

In summary, this first Polish version of the SarQoL^®^ questionnaire is equivalent to the available original version. Based on the data obtained, we have developed a new version of this valuable tool which is valid, linguistically reliable, and consistent, and therefore may be used with confidence for clinical and research purposes. The fact that it will now be available to the Polish scientific community gives physicians speaking this language the chance to better follow and monitor sarcopenic patients in our country. Thus, the Polish version of SarQol^®^ may be potentially incorporated in the routine geriatric curriculum designated for the assessment of sarcopenic Polish-speaking individuals.

## Figures and Tables

**Table 1:** Summary of subjects’ general characteristics.

	Sarcopenic (*n* = 60)	Non-Sarcopenic (*n* = 46)	*p*-Value
Age (years)	78.4 ± 8.05	71.3 ± 5.24	0.003
Sex (*n*; %) Female	43 (71.7%)	26 (56.5%)	0.10
Height (cm)	161.9 ± 9.31	164.1 ± 7.72	0.20
Weight (kg)	73.9 ± 14.2	79.9 ± 13.3	0.03
BMI (kg/m²)	28.2 ± 4.92	29.7 ± 4.91	0.12
Anthropometric data Waist circumference (cm) ^†^ Hip circumference (cm) Waist-to-hip ratio (cm) Thigh circumference (cm) ^†^	92.0 (82.2–101.5) 105.7 ± 8.37 0.87 ± 0.1 53.5 (51.0–58.0)	98.0 (89.5–108.0) 128.9 ± 131.5 0.90 ± 0.08 56.0 (53.0–62.0)	0.023 0.18 0.14 0.015
Dynamometry (kg) ^†^	19.0 (17.0–23.0)	31.0 (25.0–41.2)	<0.001
Blood pressure (mmHg) Systolic Diastolic	136.8 ± 18.1 77.8 ± 9.00	145.6 ± 22.9 79.3 ± 8.67	0.03 0.37
Marital Status Married Widowed Divorced Single	28 (46.7%) 28 (46.7%) 2 (3.3%) 0 (0.0%)	32 (69.6%) 11 (23.9%) 2 (4.3%) 1 (2.2%)	0.06
Educational Status Elementary Vocational High-school University	14 (23.3%) 10 (16.7%) 15 (25.0%) 13 (21.7%)	10 (21.7%) 8 (17.4%) 19 (41.3%) 8 (17.4%)	0.56
Number of fractures (total) ^†^	0.00 (0.00–1.00)	1.00 (0.00–2.00)	0.074
Number of falls ^†^	0.00 (0.00–1.75)	0.00 (0.00–1.00)	0.72
Number of concomitant diseases ^†^	1.50 (1.00–2.00)	1.50 (1.00–2.00)	0.33
Number of drugs ^†^	2.00 (1.00–4.00)	2.00 (1.00–4.00)	0.95

^†^ A non-parametric statistical test was used for these parameters; results are expressed as a median (P25–P75). For variables with a normal distribution, the mean ± SD is shown.

**Table 2:** Discriminative power of the Polish Sarcopenia and Quality of Life (SarQoL^®^-PL) questionnaire.

	Sarcopenia (*n* = 60) Mean ± SD	No Sarcopenia (*n* = 46) Mean ± SD	*p*-Value *
Total score	54.9 ± 16.5	63.3 ± 17.1	0.013
D1 Physical and mental health	53.7 ± 16.8	66.9 ± 19.1	<0.001
D2 Locomotion	51.3 ± 21.3	61.3 ± 22.9	0.023
D3 Body composition	53.7 ± 16.1	67.7 ± 17.8	<0.001
D4 Functionality	59.5 ± 18.4	63.9 ± 20.01	0.25
D5 Activities of daily living	53.8 ± 17.9	62.6 ± 17.8	0.014
D6 Leisure activities	27.7 ± 20.2	28.9 ± 17.3	0.75
D7 Fears	83.1 ± 11.2	86.9 ± 12.6	0.10

* *p*-value adjusted for sex, age, and systolic blood pressure.

**Table 3:** Correlations of the total score of the SarQoL^®^-PL questionnaire with its individual domains and with other quality of life (QoL) questionnaires.

	Total Score of the SarQoL^®^-PL, *r*	*p*-Value
SarQoL^®^ D1 Physical and mental health	0.91 *	<0.001
SarQoL^®^ D2 Locomotion	0.94 *	<0.001
SarQoL^®^ D3 Body composition	0.81 *	<0.001
SarQoL^®^ D4 Functionality	0.93 *	<0.001
SarQoL^®^ D5 Activities of daily living	0.92 *	<0.001
SarQoL^®^ D6 Leisure activities	0.45 *	<0.001
SarQoL^®^ D7 Fears	0.76 *	<0.001
SF-36v2^®^ PCS	0.88 ^†^	<0.001
SF-36v2^®^ MCS	0.62 ^†^	<0.001
EQ-5D index value	0.72 ^†^	<0.001
EQ-VAS	0.71 ^†^	<0.001

*r*—correlation coefficient; * Pearson’s coefficient (data of the SarQoL^®^-PL questionnaire are normally distributed); ^†^ Spearman’s coefficient (data of the SF-36v2 and the EQ-D5 questionnaires are not normally distributed); SF-36v2^®^—the Short Form-36 Health Survey questionnaire; PCS—physical component summary; MCS—mental component summary; ED-5D—the EuroQoL 5-dimension questionnaire; EQ-VAS—the EuroQoL Visual Analogue Scale.

**Table 4:** Test-retest reliability of the SarQoL^®^-PL.

	Test	Retest	ICC	95% CI
Total score	65.1 ± 13.4	65.3 ± 13.5	0.99	0.995–0.999
SarQoL^®^ D1 Physical and mental health	62.3 ± 14.4	62.0 ± 14.4	0.98	0.96–0.99
SarQoL^®^ D2 Locomotion	63.9 ± 18.6	64.8 ± 18.4	0.99	0.990–0.997
SarQoL^®^ D3 Body composition	59.9 ± 16.3	60.2 ± 15.9	0.98	0.97–0.99
SarQoL^®^ D4 Functionality	71.1 ± 14.8	70.9 ± 15.2	0.99	0.986–0.996
SarQoL^®^ D5 Activities of daily living	63.2 ± 14.3	63.5 ± 14.7	0.98	0.96–0.99
SarQoL^®^ D6 Leisure activities	40.7 ± 20.7	40.7 ± 20.7	1.00	
SarQoL^®^ D7 Fears	87.2 ± 12.1	88.1 ± 10.4	0.96	0.92–0.98

ICC—an intraclass coefficient correlation.

**Table 5:** Correlations of the total score of the SarQoL^®^ questionnaire with its individual domains: An illustrative comparison between versions published in three different languages.

	Total Score of the SarQoL^®^, *r* Polish Version	Total Score of the SarQoL^®^, *r* English Version	Total Score of the SarQoL^®^, *r* Romanian Version
SarQoL^®^ D1	0.91	0.84	0.89
SarQoL^®^ D2	0.94	0.85	0.91
SarQoL^®^ D3	0.81	0.61	0.73
SarQoL^®^ D4	0.93	0.92	0.91
SarQoL^®^ D5	0.92	0.94	0.93
SarQoL^®^ D6	0.45	0.51	0.67
SarQoL^®^ D7	0.76	0.54	0.66

*r*—Pearson’s correlation coefficient (scores are normally distributed).
